# Theranostics in Boron Neutron Capture Therapy

**DOI:** 10.3390/life11040330

**Published:** 2021-04-10

**Authors:** Wolfgang A. G. Sauerwein, Lucie Sancey, Evamarie Hey-Hawkins, Martin Kellert, Luigi Panza, Daniela Imperio, Marcin Balcerzyk, Giovanna Rizzo, Elisa Scalco, Ken Herrmann, PierLuigi Mauri, Antonella De Palma, Andrea Wittig

**Affiliations:** 1 Deutsche Gesellschaft für Bor-Neutroneneinfangtherapie DGBNCT e.V., 45122 Essen, Germany; hey@uni-leipzig.de (E.H.-H.); luigi.panza@uniupo.it (L.P.); daniela.imperio@uniupo.it (D.I.); pierluigi.mauri@itb.cnr.it (P.M.); andrea.wittig@med.uni-jena.de (A.W.); 2 NCTeam, Department for Radiotherapy, Medical Faculty, University Duisburg-Essen, 45147 Essen, Germany; 3 Neutron Therapy Research Center, Okayama University, Okayama 700-8530, Japan; 4 UGA/Inserm U 1209/CNRS UMR 5309 Joint Research Center, Institute for Advanced Biosciences, 38700 La Tronche, France; lucie.sancey@univ-grenoble-alpes.fr; 5 Institute of Inorganic Chemistry, Department of Chemistry and Mineralogy, University Leipzig, 04109 Leipzig, Germany; martin.kellert@uni-leipzig.de; 6 Dipartimento di Scienze del Farmaco, Università del Piemonte Orientale, 13100 Vercelli, Italy; 7 Departamento de Fisiología Medica y Biofísica, Universidad de Sevilla, 41004 Sevilla, Spain; mbalcerzyk@us.es; 8 Centro Nacional de Aceleradores, Universidad de Sevilla–CSIC–Junta de Andalucia, 41004 Sevilla, Spain; 9 Institute for Biomedical Technologies (ITB-CNR), 93, 20090 Segrate, Italy; giovanna.rizzo@itb.cnr.it (G.R.); elisa.scalco@itb.cnr.it (E.S.); 10 Department for Nuclear Medicine, University Hospital Essen, 45147 Essen, Germany; ken.herrmann@uk-essen.de; 11 Proteomics and Metabolomics Laboratory, ELIXIR Infrastructure, National Research Council (ITB-CNR), 20090 Segrate, Italy; antonella.depalma@itb.cnr.it; 12 Istituto di Scienze della Vita, Scuola Superiore Sant’Anna, 56127 Pisa, Italy; 13 Department for Radiotherapy and Radiation Oncology, University Hospital Jena, Friedrich-Schiller University Jena, 07743 Jena, Germany

**Keywords:** BNCT, radiation oncology, small molecules, BSH, BPA, PET, quantitative MRI, image registration, cell-penetrating peptides CPP, proteomics

## Abstract

Boron neutron capture therapy (BNCT) has the potential to specifically destroy tumor cells without damaging the tissues infiltrated by the tumor. BNCT is a binary treatment method based on the combination of two agents that have no effect when applied individually: ^10^B and thermal neutrons. Exclusively, the combination of both produces an effect, whose extent depends on the amount of ^10^B in the tumor but also on the organs at risk. It is not yet possible to determine the ^10^B concentration in a specific tissue using non-invasive methods. At present, it is only possible to measure the ^10^B concentration in blood and to estimate the boron concentration in tissues based on the assumption that there is a fixed uptake of ^10^B from the blood into tissues. On this imprecise assumption, BNCT can hardly be developed further. A therapeutic approach, combining the boron carrier for therapeutic purposes with an imaging tool, might allow us to determine the ^10^B concentration in a specific tissue using a non-invasive method. This review provides an overview of the current clinical protocols and preclinical experiments and results on how innovative drug development for boron delivery systems can also incorporate concurrent imaging. The last section focuses on the importance of proteomics for further optimization of BNCT, a highly precise and personalized therapeutic approach.

## 1. Introduction: What Is BNCT and Why Is Theranostics Necessary for the Further Development of This Modality?

Boron neutron capture therapy (BNCT) is a targeted therapy whose principle is based on the property of the isotope ^10^B to capture thermal neutrons with high probability (high effective cross-section: 3835 b), decaying into a He and a Li nucleus by the capture reaction ^10^B(n,α)^7^Li. If this reaction occurs in tissue, both particles effectively destroy cells due to their high linear energy transfer (LET) properties. Due to the short range of both particles (about 5–9 μm) in tissue, cell damage remains almost entirely confined to those cells containing ^10^B atoms. Neighboring cells containing little or no boron will not be damaged. Therefore, if ^10^B can be selectively enriched in tumor cells by suitable transport molecules, targeted destruction of malignant cells while sparing healthy tissue is possible [1].

The idea of BNCT is therefore to enable highly individualized tumor treatment, limiting the therapeutic effect exclusively to the patient-specific tumor spread. Developing this principle into a treatment option requires a compound that selectively, but with sufficiently high concentrations, accumulates ^10^B in or close to the nucleus of all tumor cells but is not toxic to healthy cells and is not toxic systemically. Both individual components of this binary treatment, namely a suitable 10B-containing compound and low energy neutrons, have little or no biological effect of their own. Only the combination of both components triggers the boron neutron capture reaction and thus leads to the therapeutic effect.

Development of this principle to a treatment modality is challenging [2] as the irradiation dose applied by BNCT in the patient, and thus the effect of BNCT, depends on the amount of ^10^B in different tissues. Even more, BNCT is not only highly precise by limiting the antineoplastic effect to malignant cells but is also highly personalized: (i) the ^10^B-uptake via a specific targeting mechanism depends on the molecular characteristics of single tumor cells, (ii) the radiation dose depends on the ^10^B concentration in single cells, (iii) spatial dose distribution depends on the subcellular ^10^B-distribution and (iv) the optimal time points of drug-application and irradiation depend on patient-specific pharmacokinetics and pharmacodynamics of the ^10^B-delivering agent (irradiation must take place at the time point when the ^10^B-concentration is highest in the tumor cells and lowest in the healthy tissue). The possibility of imaging and measuring these highly individual dependencies is thus a prerequisite for the further development of BNCT.

**Figure 1:**
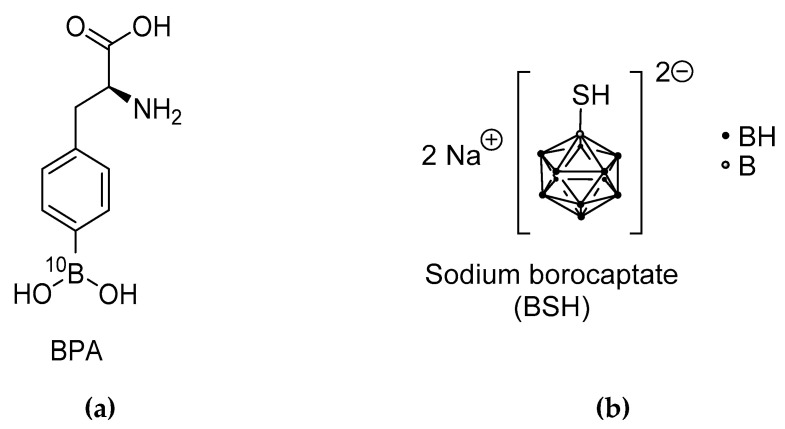
The two compounds currently used in clinical applications for BNCT (**a**) ^10^B-*p*-boronophenylalanine (BPA), (**b**) Sodium mercaptoundecahydro-*closo*-dodecaborate (BSH) [3].

At present, the ^10^B-concentration in tissue can technically not be determined at the time of irradiation. It is exclusively possible to measure the ^10^B concentration in the blood and to assume that the accumulation in the tissue, be it the tumor or organ at risk, is the same for each patient according to an experimentally determined factor. For the two compounds currently used in clinical applications for BNCT [3], namely BPA (^10^B-*p*-boronophenylalanine, C_9_H_12_^10^BNO_4_) [4] and BSH (Sodium mercaptoundecahydro-*closo*-dodecaborate, Na_2_^10^B_12_H_11_SH) [5] (Figure 1), such factors have been determined in healthy tissues, so that safe application of BNCT seems possible within narrow limits within clinical trials. However, larger controlled prospective studies are completely lacking. The well-known heterogeneity of tumor tissues in a single individual, but also between different patients, makes the accurate estimation of the dose, and therefore the prediction of the expected effect, impossible.

The recent emergence of accelerator-based facilities for BNCT placed in hospitals makes the modality independent of nuclear research reactors and thus available for routine clinical use [6]. This evolving market makes the development of new boron compounds a matter of urgency [7,8]

A theranostic approach offers the potential to overcome these challenges and to make BNCT a real precision therapy. The term “theranostics” is used for a material that combines the modalities of therapy and diagnostic imaging. A compound, which could serve as a ^10^B-delivering agent during treatment but also for diagnostic imaging of the macroscopic ^10^B-distribution and possibly allow for quantification, could enormously help further developments. Actually, this principle has been used in the therapy of thyroid tumor for the last 70 years [9]. Among the most successful examples of more recent theranostic concepts in nuclear medicine are peptide receptor scintigraphy (PRS) and peptide receptor radionuclide therapy (PRRT) for imaging and treating cancer, i.e., neuroendocrine tumors [10].

A first theranostic strategy for BNCT is already used for the compound BPA, which will be considered in more detail in Section 3 of this article.

## 2. Multi-Modal Imaging for Treatment Optimization and Response Evaluation

For several reasons, medical imaging should play a relevant role in the planning and evaluation of BNCT treatments. It can help in assessing the amount of boron compound that has reached the target site; besides, it is indispensable in creating a patient model to calculate the nuclear interactions of neutrons with tissue elements, and finally, it is necessary for evaluating the effects of the treatment on tumor and normal tissues.

Magnetic Resonance Imaging (MRI) and Computed Tomography (CT) imaging are the most-used imaging modalities for treatment planning, prescribing, and treatment response evaluation. This section is focused on the description of the current use of medical imaging for treatment optimization and response evaluation, with an overview of the future perspectives.

### 2.1. Medical Image Registration for BNCT Treatment Planning

Treatment planning in BNCT is based on the creation of a patient model containing the elemental composition of tissues within patient geometry. This is mandatory for the calculation of nuclear interactions of neutrons with tissues. For this purpose, CT images of the patients acquired before treatment are generally considered, eventually combined with MRI or PET to accurately identify the target volumes and organs at risk (OAR) [11]. Medical image registration, i.e., the estimation of a displacement vector field, is adopted in this context to realign different image acquisitions to the reference coordinate system identified by the CT and to recover spatial deformations that can occur between treatment planning and implementation of the treatment. This is particularly relevant for adaptive planning, to recalculate the delivered dose taking into account variations in geometry [12].

A typical image registration protocol adopted for BNCT treatment planning was described by Sato et al. in 2019 [13], demonstrating the influence of section thickness and field-of-view (FOV) settings on the calculated thermal and epithermal neutron flux and therefore the estimation of the dose. The need of a spatial realignment was highlighted by the non-negligible impact of patient positioning on the thermal/epithermal neutron flux estimation for dose planning. It was reported that a shift in patient positioning during treatment, but especially by set-up, has a relevant effect on dose estimation [14]. In fact, unlike conventional radiation therapies, sophisticated patient positioning tools are not available in BNCT. Furthermore, the positioning during imaging and during the treatment may be very different because the patient has to be brought directly to the rigid beam delivery system. It was found that a decrease in mean tumor dose results from high shift distances, and that dose distribution for brain tumors at greater depths would be more sensitive to patient motion [14].

The choice of the image registration algorithm depends on the imaging modalities to be realigned (mono-modal vs. multi-modal) and on the type and amount of deformation to be recovered (rigid vs. deformable registration). If the patient positioning can be accurately repeated and there are no physiological movements, a rigid registration would be sufficient in most cases, as in brain tumors; otherwise, the estimation of elastic deformation would be more appropriate. Anyway, an image registration protocol should be always optimized with respect to the anatomical district and the type of medical images available.

### 2.2. Medical Imaging for BNCT Response Evaluation: Current Use and Future Perspectives

The effects of BNCT treatment are currently evaluated using traditional imaging, such as CT, T2w-MRI and T1w-MRI before and after injection of a Gd-based contrast agent, through qualitative or semi-quantitative measures that follow the Response Evaluation Criteria in Solid Tumors (RECIST) [15]. Typical indices and radiological measures used to evaluate the tumor response to treatment are the variation in tumor volume, the enhancement area on Gd-MRI and the intensity on T2w-MRI [16,17]. With this method, treatment effects were assessed in pre-clinical rat models [18]. In patients, BNCT treatment effects were assessed in patients treated for high grade gliomas [16,17,19,20], in patients suffering from head and neck cancer (HNC) [21] and for the evaluation of adverse effects in patients treated for brain tumor [22,23]. A step toward the introduction of more quantitative image-based indices for the evaluation of treatment response was performed by Hiramatsu et al. [24], who assessed brain tumor response through the use of functional diffusion maps (fDM). This kind of approach is based on the evaluation of time-dependent variations of apparent diffusion coefficients (ADC) voxel-by-voxel, as estimated from Diffusion-Weighted MRI (DW-MRI). The inclusion of these quantitative maps has resulted in the identification of response patterns in BNCT-treated glioblastoma earlier than a standard radiographic assessment.

In conventional radiotherapy, several studies have already proposed the use of quantitative image-based biomarkers for the evaluation of treatment responses to exploit the information content of medical imaging. For example, advanced functional MRI techniques, such as diffusion- and perfusion-weighted MRI, have highlighted the high potential in determining prognosis and predicting a treatment response for high-grade gliomas [25]. The estimation of coefficients derived from mathematical models for dynamic contrast-enhanced (DCE) MRI, dynamic susceptibility contrast (DSC) MRI, and DW-MRI allows the direct quantification of diffusion and perfusion properties, that can be directly related to physiological and biological characteristics. Even more advanced techniques are available, which are based on quantitative models able to describe microstructural tissue properties, related to diffusion and perfusion, such as the Intravoxel Incoherent Motion (IVIM) [26] and Diffusion Tensor Imaging (DTI) [27]. With IVIM acquisitions, it is possible to study perfusion-related effects on DWI. The efficacy of this technique has been already established for the evaluation of response to radiotherapy for different tumors, including HNC [28]. DTI was adopted for the diagnosis, prognosis and prediction of treatment response for brain tumors [29]. The simultaneous combination of PET and MRI [^18^F]FDG PET/MRI has been shown to be superior to MRI in detecting local recurrence and metastases in patients with some special head and neck malignancies [30]. It might also offer another approach for innovative theranostics in BNCT.

**Figure 2:**
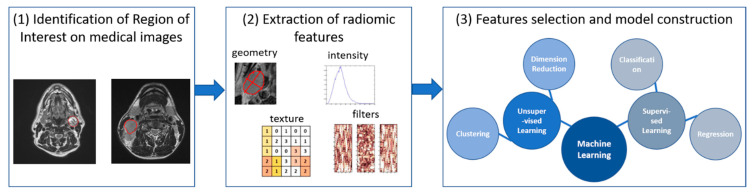
Schematic workflow of radiomic analysis. (1) Identification of Region of Interest (ROI) on medical images; (2) extraction of radiomic features (volumetric, histogram-based, texture and filter-based) within the ROI; (3) features selection and model building for prediction and prognosis.

A recent development to obtain a quantitative description of the spatial organization of tissues is the so-called radiomics, i.e., the automated high-throughput extraction of large amounts of quantitative features of medical images [31] (Figure 2). Radiomics is now largely used in oncology, revealing high potential both for tumor diagnosis and response evaluation. In addition, radiomic features can be easily associated with quantitative image-based biomarkers from perfusion and diffusion imaging, to give an exhaustive multi-parametric characterization of tissues. In this way, structural and functional properties can be considered jointly to obtain more accurate prediction models [32].

These advanced techniques may have innovative applications in the context of BNCT to evaluate the efficacy of treatment on the tumor and the impact of treatment on surrounding tissues: in addition, tumor heterogeneity estimated using radiomics could provide useful information to optimize treatment on the way to precision medicine. The previously required access to a research reactor has hampered patient recruitment for BNCT treatments and severely limited the number of controlled, prospective clinical trials. This has also hindered the adoption of advanced medical imaging techniques that have already been incorporated into the conventional radiotherapy workflow. The paucity of data has not allowed a reliable assessment of the effective improvement that quantitative image-based biomarkers might bring in this context. With the recent advent of hospital-based neutron sources for BNCT, prospective studies with larger patient cohorts can now be performed and should include evaluation of multimodality quantitative imaging to assess treatment response to provide a robust investigation of their efficacy. It also opens up the possibility of further developing the methods specifically for BNCT, which have shown promise in the early stages [33,34,35,36], for clinical use.

## 3. BPA and [^18^F]FBPA: An Existing First Theranostic Approach in BNCT

**Figure 3:**
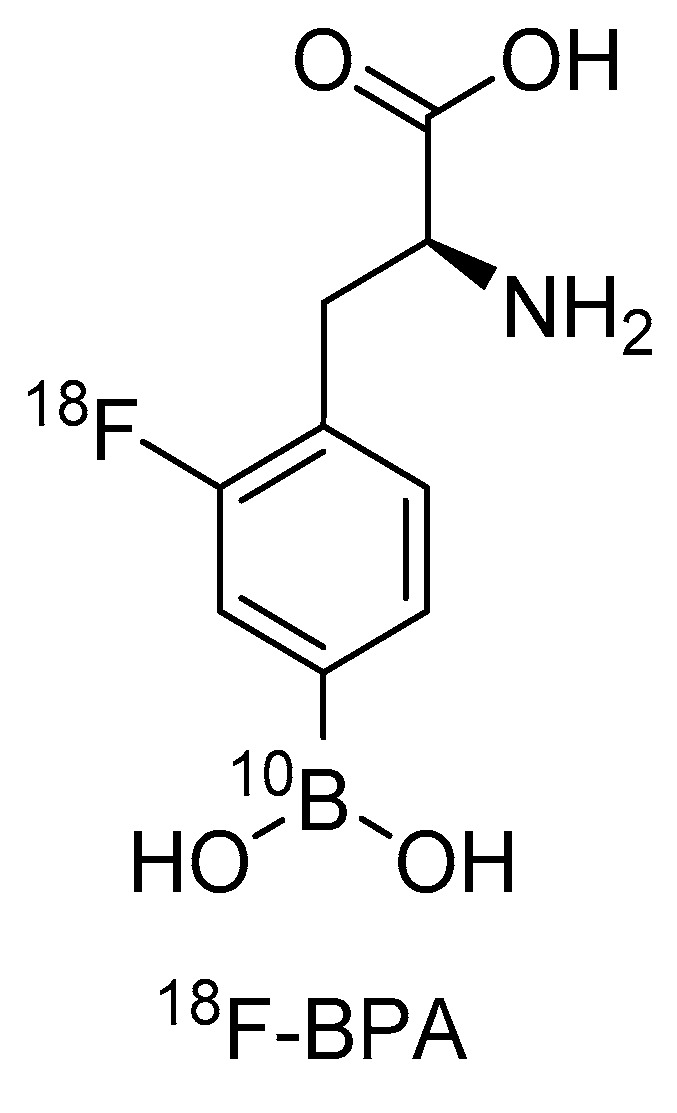
Molecular structure of ^18^F labeled FBPA (cf. Figure 1).

In this section we will focus on the use of Positron Emission Tomography (PET) as a diagnostic tool to determine the distribution of boron compounds in tissues for patient selection, treatment planning, and possible dose monitoring for BNCT using BPA as a ^10^B-compound. This drug was introduced to BNCT by Mishima in the late 1980s [37]. BPA is taken up by tumor tissues mainly by an l-type amino acid transporter (LAT-1) [38]. In 1991, Ishiwata et al. presented 4-^10^B-Borono-2-^18^F-fluoro-l-phenylalanine ([^18^F]FBPA) for monitoring the pharmacokinetics of BPA prior to BNCT [39] (Figure 3). Its use in in vivo pharmacokinetics and the quantification of BPA in patients were then performed by PET in the late 1990s by W. Kabalka et al. in the USA [40] and Y. Imahori et al. in Japan [41,42].

Imahori et al. [41] reported a method for the quantitative measurement of boronated drug uptake in patients with high-grade gliomas, based on the use of [^18^F]FBPA. A three-compartment model was used to analyze PET data and to assess the tumor pharmacokinetics. The boron concentration in the tumor calculated with the model was close to the concentration measured in surgical samples. Agreement in pharmacokinetics between l-BPA and the labeled analog was assessed using a segmental folding method. The authors concluded that the estimated values of the ^10^B concentration of BPA could be calculated from a four-rate constant model applied to a dynamic study by PET using [^18^F]FBPA as a tracer. With this approach, the ^10^B concentration was assessed prior to neutron irradiation of patients to be treated with BNCT after BPA administration. Following a similar approach, [^18^F]FBPA has been used to measure its uptake in metastatic malignant melanomas [40]; low-grade brain tumors, such as schwannoma and meningioma [43]; head and neck malignancies [44]; recurrent cancer of the oral cavity and cervical lymph node metastases [45]; and malignant glioma [46]. These early clinical findings with [^18^F]FBPA /PET led to a study of the transport and the net influx and accumulation of BPA and to show the capability of PET to screen the different types and different grades of tumor lesions as candidates for BNCT [44,45].

Currently, the use of PET/CT with [^18^F]FBPA in BNCT is to evaluate [^18^F]FBPA uptake in the tumor relative to surrounding normal tissue in individual patients and to view this information as a surrogate for the effectiveness of BNCT. Such data might be used as inclusion/exclusion criteria in clinical trials for patient selection to avoid treatment failures due to insufficient ^10^B uptake in tumors of individual patients [44]. However, as helpful as this information may be in selecting patients for BNCT, there is currently no evidence proving the correlation of uptake of [^18^F]FBPA for PET and clinical outcome after BNCT.

For various reasons, this BPA/[^18^F]FBPA approach is not yet an ideal solution for a real theranostics solution in BNCT. First of all, BPA and [^18^F]FBPA are different molecules, even though they are both transported into cells via the LAT system. More importantly, however, only μg of [^18^F]FBPA is needed for PET diagnostics, whereas several grams of BPA are applied for therapy. The resulting change in pharmacodynamics has not been sufficiently investigated and is not taken into account in the current approach. In May 2020, a BPA solution was approved as a drug (Steboronine^®^, Stella Pharma Corporation, Chuo-ku, Osaka, Japan) by the Japanese regulatory authority [47], but [^18^F]FBPA was not. Indicative pre-therapeutic diagnostics with BPA is only possible in routine operation before BPA in the frame of a clinical trial.

The design of new boron carriers for BNCT should take these aspects into account from the outset and provide atoms in suitable molecules whose non-radioactive isotopes can be replaced by a positron emitter. For therapy, a small amount of the radioactive form could be added to the real therapeutic agent and thus enable a more precise determination of the individual pharmacokinetics, the boron distribution in the body, and boron concentration in tissues during therapy.

Other radioisotopes than ^18^F might be coupled to boron-containing compounds, allowing their tracking and quantification in preclinical studies. Several gold nanoparticles were labeled with iodine (^123^I [48] or ^124^I [49,50]) for mice investigations. ^68^Ga [51] and ^64^Cu [50] may also replace the ^18^F. ^64^Cu-PLGA-micelles were investigated to vectorize ^10^B in subcutaneous melanoma with very high tumor/healthy tissue ratios, providing prolonged survival after BNCT [52]. A derivative of BSH fused with arginine peptide was labeled with ^64^Cu and showed promising brain tumor uptake, due to the addition of the arginines that facilitate the cell internalization [53].

## 4. Further Diagnostic Possibilities for Theranostic Approaches in BNCT

With the possibility of quantitative whole-body examination, PET scan is the gold standard imaging modality for monitoring and quantification of radiolabeled boron-based compounds for treatment planning, i.e., prior to BNCT. During BNCT treatment, boron concentrations are determined in the blood and extrapolated into the tumor and surrounding tissue based on prior distribution analyses. Currently, there is no real-time method to quantify boron content in an individual patient during neutron exposure.

In summary, PET is an indirect tool for monitoring the accumulation of ^10^B. The stability of the compound is important. In the design and synthesis of contrast agents, an important aspect is the desired stable covalent binding of the radioisotope or the production of a very stable complex of this isotope with the carrier molecule to prevent contamination of the patient by radioactive isotopes.

Up to now, PET-scans are used to track BPA radiolabeled with ^18^F. However, ^19^F can replace the radioisotope ^18^F, and can be observed using magnetic resonance (MR) imaging, or MR spectroscopy for blood samples. Several studies have been described on phantoms [54] or in rodents [55] for BNCT purposes. Timonen et al. reported the Finnish feasibility study in 2 patients with brain tumors, evidencing some difficulties in the localization of the [^19^F]FBPA [56] using MRI. This study should include more patients to conclude about the relevance of such investigation before and after infusion of BPA solution in patients [11].

In principle, MRI imaging and MRI spectroscopy can also be used to detect the currently available boron compounds, namely BSH and BPA, although this requires special software and hardware requirements that are not currently available for clinical use [33,34,35,36].

More generally, MRI is used to monitor the tumor volume before and after BNCT [57]. However, several preclinical studies also reported the detection of compounds containing both B and Gd or Fe as a new generation of theranostic BNCT compounds. Nanocomposites made of GdBO_3_-Fe_3_O_4_ were reported in vitro as innovative agents for magnetically targeted therapy, magnetic resonance imaging/diagnosis and Neutron Capture Therapy [58]. Recently, Alberti et al. described a Gd-chelate conjugated to carborane cages, endowed with LDL to generate a 25-nm size targeted nano-formulation able to selectively bind LDL receptors, that are overexpressed on various tumor cells, e.g., lung tumors [59] and malignant mesothelioma (MM) cells. This nano-formulation enabled them to perform MRI-guided BNCT with a very promising reduction of MM size of 85% as compared to the control group [60]. Other multimodal targeted formulations have been described to specifically accumulate in glioblastoma cells in rodents. The 90 nm size nanoparticle reported by Kuthala et al. that targets integrin α_v_β_3_ was tracked by MRI and optical imaging, allowing the prolongation of the survival of animals bearing brain tumors [61]. In preclinical stages, aza-BODIPY derivatives were investigated to enhance the vectorization of BSH in tumors. While currently visualized with optical imaging in the SWIR region, the aza-BODIPY derivatives may also be modified to add a small chelate such as DOTA moiety that could be used for nuclear imaging [62].

Ex-vivo, the quantification and the distribution of boron in tissues are also possible using elemental imaging. Such modalities might give some additional and valuable information at the cell and the tissue scales, using secondary ion mass spectrometry (SIMS) [63], laser secondary neutral mass spectrometry (laser-SNMS) [64,65], or laser-induced breakdown spectroscopy (LIBS) [62]. LIBS is an all-optical technique working at room temperature and atmosphere, allowing the reconstruction of elemental imaging from the mapping of a sample. In LIBS, the laser-induced plasma generated by focusing laser pulses on the surface of the sample allows a specific optical response to be elicited from the elements constituting the sample. This specific response is collected using an optical spectrometer before the reconstruction in a pixel-by-pixel manner of the map [66,67,68]. A real sub-cellular detection of ^10^B in cells can be achieved by electron energy-loss spectroscopy (EELS) [69,70]. A well-established method for mapping ^10^B distribution in organs is neutron autoradiography [71,72]. Wittig et al. summarized these methods as applied to BNCT in 2008 [73].

## 5. Molecular Modalities with Theranostic Applications in BNCT

### 5.1. Small Molecules

For cancer treatment, it is necessary to understand and investigate the dimension and various other properties of the malignant tissue to adapt the planned therapy. Especially in BNCT, a very important aspect is the detection, correct localization and quantification of ^10^B in diseased tissues to guide radiotherapy treatment. Therefore, the development of small molecules that can be used for therapeutic and diagnostic purposes in vivo, so-called theranostic agents [74,75,76], is highly important.

Based on this dual approach, a variety of potential small-molecule BNCT agents was conjugated with an imaging agent. For therapeutic applications, the respective drug should act as a tumor-selective compound and allow high accumulation of boron in cancer cells, as well as exhibit all other properties required for BNCT agents [77].

For diagnostics, mainly PET and MRI are employed for in vivo imaging.

**Figure 4:**
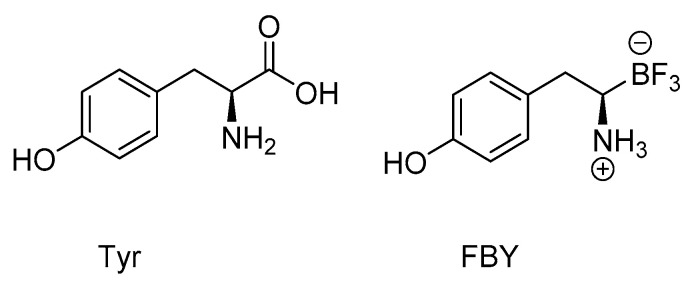
Molecular structures of Tyr and FBY in comparison.

The radiolabeled compound [^18^F]FBPA (see paragraph 3) (Figure 3) is the most-used BNCT–PET theranostic drug [78]. Several other small molecules like [^18^F]FBPA can be used in this approach, such as the modified boron-derived tyrosine, fluoroboronotyrosine FBY (Figure 4), reported in 2019. FBY, a tyrosine analogue in which a COOH group is replaced by a BF_3_ moiety, was proposed as a theranostic agent [79]. FBY showed superior chemical stability compared with BPA, a high radiochemical yield of 50% and a high radiochemical purity of 98%. Its similarity with the natural amino acid tyrosine enables uptake via the LAT-1 system, and thus, accumulation of up to 128 µg FBY/10^6^ cells. The possibility of non-invasive imaging was indicated by the administration of [^18^F]FBY to mice followed by PET imaging. Furthermore, FBY shows no obvious systemic toxicity, which renders this compound a very promising theranostic agent for image-guided BNCT [79]. A small preclinical study with six animals comparing BPA and FBY showed slightly better results for FBY in terms of animal survival and tumor size 3 weeks after BNCT with 1 g/kg intravenous injection of FBY and BPA and 30 min of 10^12^ n/(cm^2^ s) neutron flux. The tumor was placed in the right shoulder of the mouse. No kinetic data on FBY were presented [79].

**Figure 5:**
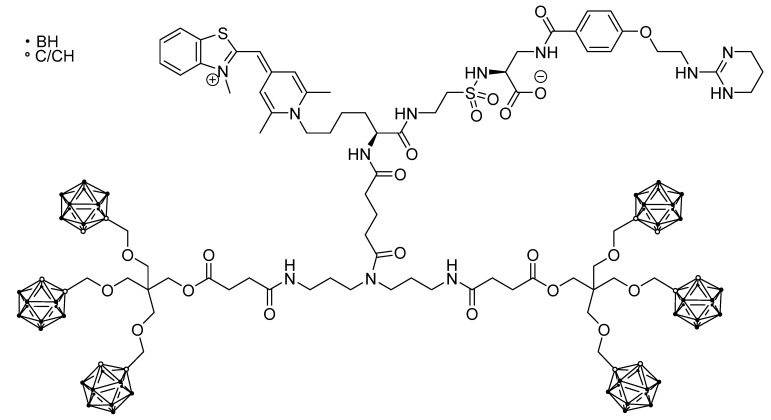
Dumbbell-shaped molecule combining carboranes, a nonpeptidic RGD- mimetic α_v_β_3_ integrin ligand and a monomethine cyanine dye for fluorescence imaging [80].

Another imaging method that is, however, mainly applied in vitro, is fluorescence imaging. One theranostic agent that was also employed for in vivo imaging was a dumbbell- or H-shaped molecule bearing six carborane units, a nonpeptidic RGD-mimetic α_v_β_3_ integrin ligand for tumor-targeting and a monomethine cyanine dye for fluorescence imaging (DC-1, Figure 5). This compound was shown to accumulate in MCF7 (breast cancer) and WM115 (melanoma) cells exploiting the α_v_β_3_ integrin targeting unit. Visualization of the uptake was achieved by in vivo fluorescence spectroscopy and intravital microscopy (IVM) [80]. Although this compound is a single molecule, the molecular weight of around 1.2 kDa would not allow it to be classified as a small molecule [81].

### 5.2. Cell-Penetrating Peptides (CPP) for BNCT

CPPs are short peptides (less than 30 amino acids) that have been predominantly used in basic and preclinical research over the last 30 years. They are not only capable of translocating themselves into cells but also facilitate “cargo” moieties ranging from nanosized particles to small chemical compounds to translocate across the plasma membrane mainly through various endocytosis mechanisms. The cargo is joined to CPPs either by a covalent bond or by non-covalent interactions [82]. Several authors were investigating if CPPs could be applied as a delivery system to carry boron compounds into cancer cells in vitro and in vivo for BNCT [83,84,85,86].

The research was focused on BSH (Figure 1), being highly desirable for BNCT compounds containing multiple boron atoms, which makes the delivery of ^10^B more efficient. However, this compound has a significant drawback as it is not able to easily cross the cell membrane directly, due to the negative charge of the boron cluster. To overcome this problem, an oligoarginine peptide was conjugated to a dendritic lysine bearing multiple BSH moieties. The structure containing eight BSH units was tested in vitro on U87 glioma cells in comparison with BSH alone and other BSH-CPPs conjugates showing an impressive uptake. Studies in vivo on the same U87 cells injected into the striatum of nude mice demonstrated that the compound accumulated into the tumor but was not detected in the normal brain [85].

In a quite different approach, a series of palmitoyl peptides known to be able to flip across the membrane to penetrate cells were bound to BSH, and among them two structures were identified that effectively accumulate boron within T98G glioblastoma cells [83].

**Figure 6:**
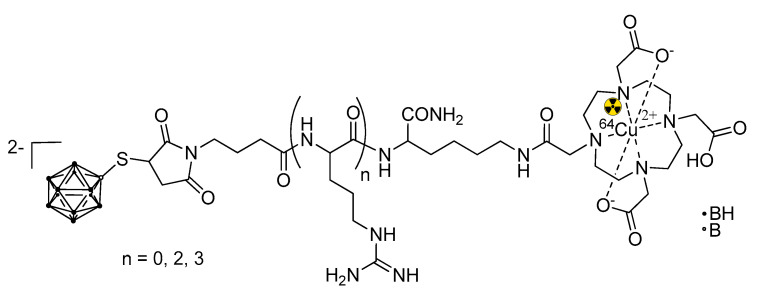
A theranostic BNCT construct based on CPPs [53]. The trefoil symbol at ^64^Cu indicates the radioactive element of this molecule.

A BSH fused with a short oligoarginine domain was conjugated with 1,4,7,10-tetraazacyclododecane-1,4,7,10-tetraacetic acid (DOTA) and labeled with positron emitter ^64^Cu (Figure 6). Positron emission tomography was allowed to follow the pharmacokinetics of the compound in vivo in a U87δEGFR brain tumor bearing mice [53]. It is worth noting that this is the only example of a theranostic BNCT construct based on CPPs. To date, there are only a few examples of ^18^F labeling of CPPs [87], but none in the BNCT field.

The same group from the Neutron Therapy Research Center at Okayama University exploited another BSH-oligoarginine construct showing its binding to the CD44 cell-surface molecule for cellular uptake [88].

Organelle-targeted cell-penetrating peptides with high plasma membrane permeability, conjugated with boron cluster BSH, were synthesized and tested. The products were designed to control their location within the cells, in particular, to target mitochondria and endosomes. To follow the intracellular distribution, the constructs were labeled with a fluorescent probe. The study allowed us to visualize specific subcellular distributions of conjugates bearing different CPPs. Moreover, upon irradiation with thermal neutrons, one of the products showed a high cell-killing activity. Investigation on the cell death pathways revealed an increase in markers of cell apoptosis and a decrease in ATP content, thus contributing to an understanding of the mechanisms involved in cell death when submitted to BNCT treatment [86].

### 5.3. Larger Boron Vectors: Proteins, Antibodies and Nanoparticles

Larger boron-carriers as proteins, epidermal growth factors (EGF), antibodies (monoclonal antibodies; mAbs) and nanoparticles were created for increased selectivity and delivery of a large amount of ^10^B. Due to their size, these compounds facilitate the radiolabeling using ^18^F, ^123^I, ^111^In, ^64^Cu, ^68^Ga, and ^177^Lu for nuclear imaging, or the addition of Gd for MRI investigations.

Albumin is widely used in oncology as a single molecule and nanoparticle carrier [89], as it accumulates in tumor lesions and inflamed tissues through the enhanced permeability and retention (EPR) effect, and possesses multiple cellular receptors and ligand binding sites. This abundant protein (35–50 mg/mL in human blood) possesses hydrophobic binding pockets facilitating the transport of hydrophobic molecules. Different strategies can be evaluated for efficient tumor delivery, including nanoparticle formulation, covalent conjugation, and genetic fusions. In BNCT approaches, covalent conjugation of a maleimide-*closo*-dodecaborate on albumin lysin (Lys221, Lys413 and Lys431) and cysteine Cys34 residues was reported to selectively accumulate in colon-26 tumor-bearing mice; such a molecule, when used as a boron carrier for BNCT, demonstrated a strong tumor volume reduction at low dose (7.5 mg[B]/kg) [90].

Another biological target, the epidermal growth factor receptor (EGFR), is over-expressed on high-grade glioma, a tumor type that might be treated with BNCT. Boron-rich compounds, involving a boronated starburst dendrimer (BSD), have been conjugated to mAb anti-EGFR. The boronate-mAb conjugate (BD-C225) demonstrated a specific accumulation in tumor cells expressing the EGFR in vitro. In vivo, intratumoral injection [91] and convection-enhanced delivery (CED) administrations [92] demonstrated prolonged and favorable tumor retention at 24 h (>50 μg/g for CED in tumor expressing the EGFR), with tumor/brain ratio ≈10. Interestingly, when BNCT was performed 24 h after CED administration of BD-C225, the mean survival time (MST) reached 54.1 days and increased to 86 days in combination with i.v. administration of BPA (500 mg/kg) [93]. As a control, the MST of animals irradiated without drugs was 30.9 days. Altogether, these studies demonstrated the potential of targeted molecules and the effectiveness of combined treatment with the clinically approved BPA.

In these studies, the boron carriers were not quantified in vivo using imaging, but ex-vivo quantifications were reported to optimize BNCT efficacy; however, both albumin [94] and anti-EGFR [95] can be labeled for imaging and treatment planning.

**Figure 7:**
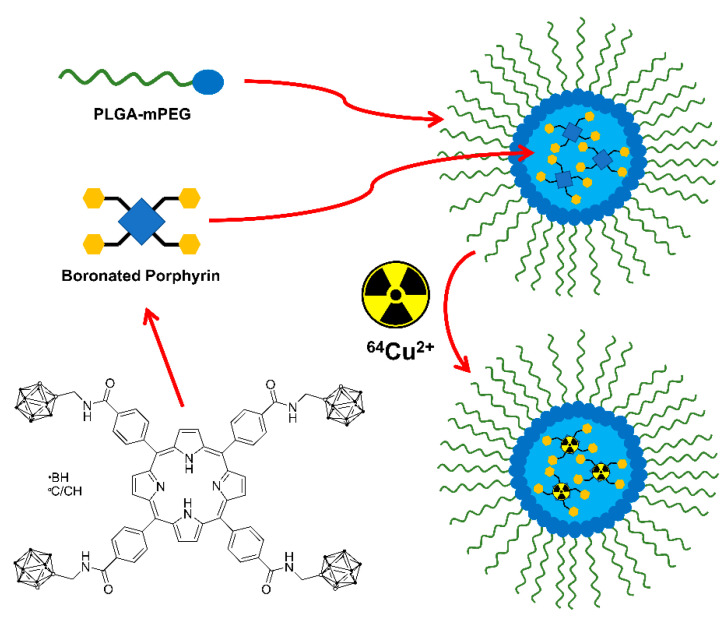
^64^Cu-containing boronated porphyrin incorporated into a biocompatible polymer nanoparticle [52]. The trefoil symbol at ^64^Cu indicates the radioactive element of this molecule.

**Figure 8:**
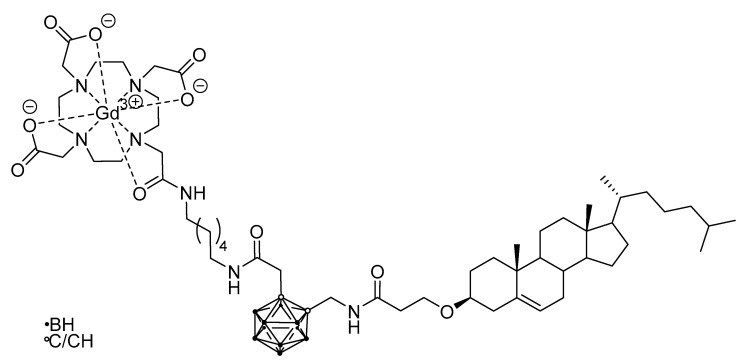
Gd^III^- and carborane-containing cholesterol derivative for application as a liposome precursor with MRI imaging abilities [96].

In other cases, nanoparticular entities were employed as the tumor-selective vehicle, like a biocompatible polymer nanoparticle carrying a boronated porphyrin complex with a ^64^Cu isotope [52] (for PET, Figure 7), or a liposome for a carborane-containing cholesterol derivative bearing a Gd^III^ complex (for MRI, Figure 8) [96].

**Figure 9:**
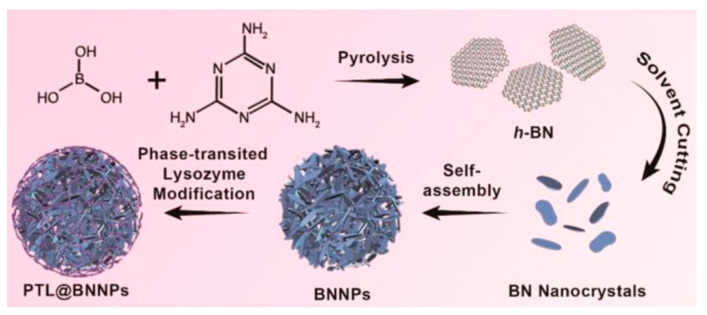
Schematic representation of the preparation of BNNPs coated with phase-transited lysozyme. Reprinted with permission from Li L et al. ACS Nano. 2019;13(12):13843-52 [97]. Copyright (2019) American Chemical Society.

Boron nitride nanoparticles coated with phase-transitioned lysozyme (PTL@BNNPs) to induce in vivo degradation upon i.v. injection of vitamin C were prepared as BNCT agents (Figure 9) [97]. Cellular uptake in 4T1 cells reached 200 ppm boron (10 mM). These nanoparticles were labeled with a PET radioisotope, namely ^64^Cu, in a chelator-free method by simply mixing ^64^CuCl_2_ with the PTL@BNNPs in 0.1 M hydrochloric acid, transfer into a sodium acetate buffer solution and subsequent ultrafiltration for purification prior to theranostic use. The BNCT treatment was effective in a small group of animals (n = 6) with 4T1 shoulder-inoculated tumor. The animals survived 3 weeks; however, the tumor kept growing.

**Figure 10:**
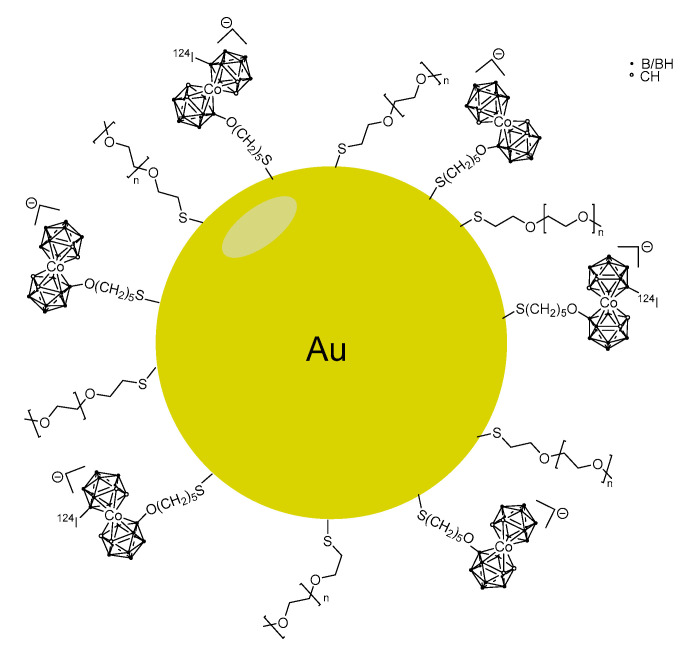
Example of a cobalt bis(dicarbollide)-modified gold nanoparticle with ^124^I-labelling on the carborane periphery.

Gold nanoparticles (NP) coated with the boron-rich cobalt bis(dicarbollide) anion and stabilized with polyethylene glycol were synthesized (Figure 10) [49]. The NP were successfully labeled with ^124^I, on the NP surface and in the cobalt bis(dicarbollide) periphery (4.18 days half-life) for PET imaging. The incorporation in the mouse model of human fibrosarcoma (HT1080 cells) reached 0.06 mM, which is too low for BNCT. On the other hand, biodistribution studies in a xenograft mouse model of human fibrosarcoma showed major accumulation in the liver, lungs and spleen. These results indicate the necessity of further optimization in terms of shape and size of the gold nanoparticles and pre-targeting might be an option [98].

Recently, Feiner et al. [98] investigated a pre-targeting strategy using ultra-small nanoparticles (boron-rich carbon dots) to reduce off-target side effects caused by long circulations times during application of normal nanocarriers. They describe the preparation, characterization and in vivo evaluation of tetrazine-functionalized boron-rich carbon dots. These particles show a very fast clearance from blood. Tumor accumulation was achieved by using a pre-targeting approach, which was accomplished by a highly bioorthogonal reaction at the tumor site with *trans*-cyclooctene-functionalized Trastuzumab. The authors also provide an outlook to transfer the explored methodology to other pre-targeting-based therapeutic approaches to decrease off-target side effects, e.g., in radionuclide therapy. Modifications in the protocol of the presented methodology provide a basis for the development of new therapeutic strategies, which may improve the efficacy of already available therapeutic approaches on the market.

## 6. Proteomics for Predicting Outcome

In the last two decades, genomics, including the analysis of both genome and transcriptome, has gained a strong impulse in translational research. These methodologies have the potential to lead to highly innovative and ambitious projects with great added value for the understanding of disease development and the effects of therapeutic interventions. Proteomics will become increasingly important for theranostics in the short term and should therefore not go unmentioned here.

The main goals of high-throughput molecular approaches, also called “Omics Technologies”, concern:

- discovery and validation of biomarkers useful for diagnosis and follow-up of therapy;
- characterization of metabolic pathways involved in the investigated diseases;
- elucidation of molecular mechanism (endotypes) related to disease etiology and clinical stratification.

Omics Technologies, combined with computational tools, are permitting the development of system-oriented approach, called Systems Biology, or Systems Medicine when applied to clinical fields [99].

Although genes are of primary importance for life, they are not sufficient to explain the environmental effects that determine phenotypes. It is well known that the genome is not completely static; however, proteins and metabolites, as molecular effectors of genes, are more sensitive to various stimuli and they amplify disease-related impairments. Moreover, due to alternative splicing and post-translational modifications (PTMs), multiple proteins are associated with a single gene or mRNA. In fact, it is estimated that there are about 25,000 genes, 40,000 unique metabolites, about 100,000 mRNA transcripts, and more than a million different protein isoforms.

For these reasons, it is imperative to move beyond the predominant study of the genome/transcriptome and enhance the study of the proteome and metabolome to understand the molecular basis of disease as a prerequisite for accurate diagnosis and effective therapy.

Improvements in mass spectrometry and chromatography technologies, which form the basis of high-throughput proteomic approaches, are enabling improvements in the precision of proteomic profiles and reductions in the amount of sample required for analysis. This will enable overcoming the use of the pooled samples, which is a mandatory requirement for truly personalized medicine [100]. Shotgun proteomics is quantitative, based on different methods to evaluate the differential expression of proteins comparing different conditions [101], and it is possible to analyze a wide range of samples: cell lines, biofluids and tissues, both fresh, frozen or paraffined; in particular, the possibility to analyze formalin fixed paraffin embedded (FFPE) samples improves the availability of human samples useful for proteomic analysis.

The availability of samples collected from patients is one of the most important critical issues. In principle, biopsies of tissues where disease is localized, and where molecular signatures are more common and specific are the best samples for studying human diseases. However, collection of disease-relevant tissues is not always easy or possible, e.g., in the brain. This necessitates the development of methods to study samples obtained without invasive procedures, such as cell lines and biofluids (mainly blood or urine), to identify secreted proteins as potential biomarkers of physio-pathological conditions. Three-dimensional cultures, called organoids, also represent good models that approximate actual cancer tissues.

Biofluids are usually easy to collect. They contain the potential disease-related signals, but these signatures are highly diluted or overloaded by large amounts of a few proteins, mainly albumin and immunoglobulin in plasma, compared to samples in diseased tissues. It is possible to separate the signals from the baseline biochemical noise by isolating circulating microvesicles (mEVs), also called exosomes. They are considered carriers, containing various molecules such as metabolites, RNAs, and proteins and they transport the secreted signals (secretomes) from specific tissues, comparable to a “message in a bottle”. Clearly, mEVs are very interesting for cancer diagnosis and monitoring, as they could reflect changes in damaged or recovered tissues without invasive intervention.

Another important issue of clinical proteomics is the identification of the pathways dysregulated in the disease; because it is not a single protein changed in the disease, but a cluster of interacting proteins, representing a pathway. For this reason, the large amount of data generated by shotgun proteomics can be processed with computational tools to correlate protein profiles to biological processes, matching clinical phenotypes and proteomics data by means of the Systems Biology strategy, and improving an actual Personalized Medicine.

Proteomics are widely used to investigate both tumor biomarkers and endotypes, such as the role of energy metabolism [102,103]. In contrast, few applications have been related to boron neutron capture therapy (BNCT). Sato et al. investigated by gel-based proteomics the early cellular responses after BNCT on human squamous cell carcinoma SAS cells incubated with BPA prior to neutron irradiation [104]. The results suggest that LRMPs (lymphoid-restricted membrane proteins) are specific for BNCT and may play a physiological role in the process of BNCT-induced cell death. Recently, Ferrari et al. used a gel-free approach to characterize the protein profiles of urinary exosomes from patients with head and neck squamous cell carcinoma and thyroid cancer who had received BPA and BSH, respectively, prior to tumor resection in an early clinical trial [105]. Both BPA and BSH treatment were shown to reduce the levels of proteins associated with inflammation and tumor progression processes, including CD44, galectin-3-binding protein, and osteopontin. Finally, BSH was shown in vitro to bind proteins via cysteines [106].

In particular, BNCT, as an innovative tumor-targeted therapy, can take advantage of the methodological setups of molecular profiling to improve the understanding of the effects of novel boron-containing compounds and their combination with neutron radiation. Subtyping of patients eligible for a beneficial effect of BNCT treatment thereby becomes possible. This will reduce costs by avoiding ineffective therapies facilitating the selection of the optimal therapeutic modality for a specific patient, which is the ultimate goal of personalized medicine.

In particular, with the use of non-invasive samples, it is possible to study the precise molecular profiles and characterization of disrupted signaling pathways in relation to the disease and to evaluate both the efficacy and side effects of therapy.

Although molecular profile approaches, mainly those such as proteomics and metabolomics, do not exactly correspond to the definition of theranostic activities, they allow a high precise diagnostic typing and personalized prediction of treatment results [107]. In particular, system biology investigations based on proteomics are able to elucidate the endotypes related to pathogenesis and/or the positive effect of treatment. Recently Rainone et al. [108] have used proteomics to demonstrate the specificity and efficacy of targeted nanoparticles as a new theranostic agent. In this context, it is possible to include in the theranostic activities the availability of proteomics to analyze non-invasive samples and to characterize the molecular targets related to tumor aggressiveness, beyond the small molecules.

Clearly, the actual use of proteomics to improve BNCT for developing theranostic approaches will require a close combination of techniques from very different disciplines, such as organoids and 3D bioprinting for ex vivo experiments, and innovative imaging, including data processing evaluations (see radiomics in Section 2 of this article). In the future, the combination of all the generated data will allow clinicians to obtain a more detailed profile of an individual patient to further individualize precision medicine.

## 7. Conclusions

BNCT is a treatment modality of precision medicine [109,110]. Its success depends on the optimized customization of dose applications to a given individual patient. To fully exploit the potential of BNCT, one challenge is to use omic methods to identify biomarkers that allow the definition of subgroups of patients for whom specific targeting ^10^B-carriers are appropriate. The approach with ^18^F-labeled BPA described in Section 3 of this article is a first step in addressing this issue. However, this approach is not sufficient because it does not capture the heterogeneity of tumors and the individual dynamics of the uptake of boron carriers into target cells. To make optimal use of BNCT, ways must be found to evaluate the boron concentration in the tumor and the organs at risk as precisely as possible in direct temporal relation to the irradiation. The principles of theranostics offer the possibility to achieve this goal. New boron carriers should be designed a priori so that their distribution in the body and their concentration in specific tissues can be determined directly before (or even during) irradiation.

Research efforts are underway to identify new candidates for BNCT that have a better tumor selectivity, a longer cell retention, and whose accumulation in the patient’s internal organs can be monitored by various techniques, with a focus on PET. The 4-^10^B-Borono-2-^18^F-fluoro-l-phenylalanine ([^18^F]FBPA) was the first compound developed for monitoring the pharmacokinetics of 4-^10^B-borono-phenylalanine, which is still used in BNCT. MRI can be alternatively used to monitor the tumor before and after BNCT treatment and different preclinical studies have reported the detection of compounds containing both B and Gd or Fe as a new generation of theranostic BNCT agents. Fluorescence imaging mainly applied in vitro found an application in BNCT in vivo with a nonpeptidic RGD-mimetic integrin ligand and a cyanine dye. An alternative approach is the conjugation of BSH with CPPs. When BSH, linked with a short oligoarginine peptide, was conjugated with 1,4,7,10-tetraazacyclododecane-1,4,7,10-tetraacetic acid (DOTA) and labeled with a positron emitter ^64^Cu, the monitoring of the compound in vivo in U87ΔEGFR brain tumor-bearing mice by PET was possible. Proteins, growth factors, antibodies, and nanoparticles were conjugated to increase the selectivity and delivery of a large amount of ^10^B. When a maleimide-*closo*-dodecaborate was linked on albumin lysin and cysteine Cys34 residues, the compound was selectively accumulated in colon-26 tumor-bearing mice. The boronate-mAb conjugate (BD-C225) demonstrated a specific accumulation in tumor cells expressing the EGFR in vitro. Nanoparticular entities (i.e., boronated porphyrin complex with a ^64^Cu isotope or a liposome for a carborane-containing cholesterol derivative bearing a Gd^III^ complex) were used without satisfying tumor uptake for BNCT. These results indicate the need for further optimization of the shape and size of the gold nanoparticles. Moreover, a future approach will involve Omics Technologies, combined with computational tools allowing the development of the so-called Systems Medicine. In particular, BNCT could take advantage of the methodological setups of molecular profiling to improve the understanding of the effects of novel compounds and their combination with neutron radiation. The most relevant aspect emerging from the work here described is the increasing effort towards new theranostic agents that may help to create breakthroughs in the BNCT field.
